# Rapid obtention of stable, bioluminescent tumor cell lines using a tCD2-luciferase chimeric construct

**DOI:** 10.1186/1472-6750-11-26

**Published:** 2011-03-24

**Authors:** Anne-Sophie Jimenez, Mélanie Gressette, Clément Barjon, Ming Wei, Claire Gourzones, Pierre Busson

**Affiliations:** 1 Université Paris-Sud 11, CNRS-UMR 8126 and Institut de Cancérologie Gustave Roussy, 39 rue Camille Desmoulins, 94805 Villejuif cedex, France; 2 Cellvax, Bâtiment Marcenac, aile Est, Ecole Nationale Vétérinaire d'Alfort, 7 avenue du Général de Gaulle, 94704 Maisons Alfort cedex, France

## Abstract

**Background:**

Bioluminescent tumor cell lines are experimental tools of major importance for cancer investigation, especially imaging of tumors in xenografted animals. Stable expression of exogenous luciferase in tumor cells combined to systemic injection of luciferin provides an excellent signal/background ratio for external optical imaging. Therefore, there is a need to rationalize and speed up the production of luciferase-positive tumor cell lines representative of multiple tumor phenotypes. For this aim we have designed a fusion gene linking the luciferase 2 protein to the c-terminus of a truncated form of the rat CD2 protein (*tCD2-luc2*). To allow simultaneous assessment of the wild-type luciferase 2 in a context of tCD2 co-expression, we have made a bicistronic construct for concomitant but separate expression of these two proteins (*luc2-IRES-tCD2*). Both the mono- and bi-cistronic constructs were transduced in lymphoid and epithelial cells using lentiviral vectors.

**Results:**

The tCD2-luc2 chimera behaves as a type I membrane protein with surface presentation of CD2 epitopes. One of these epitopes reacts with the OX34, a widely spread, high affinity monoclonal antibody. Stably transfected cells are sorted by flow cytometry on the basis of OX34 staining. *In vitro* and, moreover, in xenografted tumors, the tCD2-luc2 chimera retains a substantial and stable luciferase activity, although not as high as the wild-type luciferase expressed from the *luc2-IRES-tCD2* construct. Expression of the tCD2-luc2 chimera does not harm cell and tumor growth.

**Conclusion:**

Lentiviral transduction of the chimeric *tCD2-luc2 *fusion gene allows selection of cell clones with stable luciferase expression in less than seven days without antibiotic selection. We believe that it will be helpful to increase the number of tumor cell lines available for *in vivo *imaging and assessment of novel therapeutic modalities. On a longer term, the tCD2-luc2 chimera has the potential to be expressed from multi-cassette vectors in combination with various inserts of interest.

## Backround

Most recent developments in therapeutics against cancers have underlined the need to adapt treatment modalities to the molecular and functional characteristics of each tumor. For example, antibodies targeted to the EGF-receptor provide benefits only for a subset of colon carcinoma with K-ras mutations [1]. Therefore, for each tumor type, there is a need for investigations of novel therapeutic arms on a large number of tumor cell lines representative of various genotypes and phenotypes. This requirement applies to *in vitro *but also *in vivo *investigations which are a necessary step for evaluation of novel therapeutic modalities. To perform these *in vivo *explorations, optical imaging is an irreplaceable tool to assess dissemination of tumor cells in the body of xenografted mice as well as tumor growth or regression under treatment [2,3]. Although various types of reporter genes encoding fluorescent proteins have been reported, expression of exogenous luciferase combined to systemic administration of luciferin remains the strategy of optical imaging providing the best signal/background ratio [4].

Currently luciferase-positive tumor cell lines are often produced by transfection of a plasmid containing a luciferase expression cassette and a gene encoding antibiotic resistance as a selection marker. This approach has two major drawbacks: it often requires several weeks of manipulation and the process of antibiotic selection is a factor of uncontrolled phenotypic changes. To overcome these problems, we have designed a strategy based on a chimeric protein in which the luciferase 2 is fused to the C-terminus of a truncated form of the rat CD2 protein. As reported here, this protein behaves as a type I membrane protein which retains luciferase activity whereas its CD2 portion is presented at the surface of the plasma membrane. Lentiviral transduction of the chimeric gene combined to flow cytometry sorting of CD2-positive live cells allow rapid production and selection of luciferase-positive cells derived from both lymphoid and epithelial tumor cells.

## Methods

### Construction of plasmid inserts containing the luciferase 2 gene

A fusion gene named *tCD2-luc2 *was assembled in the pcDNA6.2/V5/GW/D-TOPO (Invitrogen, Cergy Pontoise, France). First, a cDNA segment encoding for the first 232 residues of the rat *CD2 *(GenBank ADI96088.1) was PCR-cloned into this plasmid with addition of an EcoR1 site at the 3' end of the insert. In a second step, the full length gene encoding *luciferase 2 *(*Photinus pyralis*) was PCR-amplified from pGL4.10 (Promega, Charbonnières-les-Bains, France) with addition of EcoR1 sites at both ends. Some codons of this synthetic *luciferase 2 *have been optimized for more efficient expression in mammalian cells (GenBank AAV52869.1). In the third step, the *luc2 *gene was ligated in the EcoR1 site downstream of the truncated *CD2 *gene (T4 ligation). This construct resulted in a chimeric gene containing the first 232 codons of the rat *CD2*, 2 codons corresponding to the EcoR1 site in frame with the full length *luc2 *gene, under the control of the CMV promoter. The pairs of primers designed for cloning of the *CD2 *and *luc2 *segments are presented in Table 1. A bicistronic construct containing the full length *luciferase 2 *and the truncated rat *CD2 *genes, was assembled in the pQCXIX plasmid (Takara Bio Europe/Clontech, Saint-Germain-en-Laye, France). First, the *luciferase 2 *gene was PCR-amplified from pGL4.10 (Promega) with addition of Not1 sites at both ends and an internal Mlu1 site at the 5' end. The truncated rat *CD2 *gene was PCR-amplified with addition of Bcl1 sites at both ends and an internal Mlu1 site at the 3' end. Then, these two genes were inserted into the MCS I (at the Not1 site) and MCS II (at the Bcl1 site), respectively. They were thus linked by the IRES (Internal Ribosome Entry System) provided by the pQCXIX plasmid. All PCR reactions were done using Finnzymes' Phusion™ Hot Start High-Fidelity DNA Polymerase (Ozyme, Saint Quentin, France). Final constructs were verified by sequencing.

**Table 1:** Primers used to amplify DNAs coding for a truncated form of rat CD2 (codons 1-232) and the full-length luciferase 2

Sequences to amplify	Forward primers	Reverse primers
*rat tCD2*	5'CACCATGAGGTGTAAATTCCTAGGG3'	5'GAATTCCCGTTTTTTCCTCTTGCAGAT3'
*luc 2*	5'CACCGAATTCATGGAAGATGCCAAA3'	5'GAATTCTTACACGGCGATCTTG3'

### Construction of expression vectors for short-term transfections

As explained in the previous paragraph, the *tCD2-luc2 *fusion gene was assembled directly in the pcDNA6.2/V5/GW/D-TOPO (Invitrogen). To allow comparison of the chimeric protein with the wild-type luciferase 2 in short-term transfections, the wild-type *luc2 *gene was PCR-cloned in the same vector at its EcoR1 site.

### Construction of the pro-lentiviral vectors and production of lentiviral particles

The two inserts of interest - *tCD2-luc2 *and *luc2-IRES-tCD2 *- were cloned in the pLV plasmid (Vectalys SA, Toulouse, France). This pro-lentiviral plasmid provides an expression cassette driven by the elongation factor-1 alpha (EF-1 alpha) promoter. This promoter was chosen for its steady, mild transactivation power which is consistent *in vitro *and *in vivo *[5]. The pLV expression cassette also contains at its 3' end the woodchuck hepatitis virus post-transcriptional regulatory element (WPRE). WPRE was shown to stimulate expression of intronless viral messages not only in cells infected by woodchuck hepatitis virus, but also in nonviral and heterologous viral gene delivery systems [6]. Finally the pLV expression cassette is surrounded by HIV LTRs deleted of the U3 region [pLV-EF1-tCD2-luc2-WPRE] (Figure 4). The *tCD2-luc2 *insert was PCR-cloned at the Mlu1 site of the pLV vector. The *luc2-IRES-tCD2 *insert was excised from the pQCXIX plasmid where it was previously assembled by an Mlu1 cut and ligated at the Mlu1 site of pLV. Lentiviral particles were produced by Vectalys SA. Pro-lentiviral plasmids derived from pLV and containing appropriate inserts were co-transfected in 293T cells along with two helper plasmids encoding for the *gag *and *pol *HIV genes and the *VSV-G Env *gene, respectively.

### Cell culture and transfections

The epithelial (293, HeLa and MDA-MB-231) and lymphoid cells (Daudi) were grown in DMEM and RPMI 1640 medium, respectively (Gibco-Invitrogen, Cergy Pontoise, France) with 10% fetal bovine serum (FBS).

For short-term transfections, 293 cells were seeded in six-well plates at 2.10^5^cells/well. On the next day, they were transfected with increasing concentrations of plasmid DNA encoding for the wild-type luciferase 2 (luc2) or the tCD2-luciferase 2 (tCD2-luc2) chimera (from 0.05 μg to 1 μg/well i.e. 25 ng to 500 ng/10^5^cells). Transfection was performed using TurboFect™ *in vitro *Transfection Reagent (Fermentas, St. Leon-Rot, Germany) according to the manufacturer's instructions. Cells were grown *in situ *for 48 h prior to cell counts and protein extraction for luciferase assay and western blot analysis.

Stable transfections in Daudi and Hela cells were made by lentiviral infection. Target cells were incubated with recombinant lentiviral particles (multiplicity of infection 2) in the presence of hexadimethrine bromide 4 μg/mL (Sigma-Aldrich, St Quentin Fallavier, France) to enhance transfection efficiency [7]. Forty-eight hours later, transduced cells were extensively washed and subjected to flow cytometry sorting on the basis of OX34 staining.

### Luciferase assay

Luciferase activity in cell extracts was assessed using the Promega Luciferase Assay System according to the manufacturer instructions (Promega). Washed cells were lysed using the lysis reagent provided in the kit. The Plate-Reading luminometer's injectors added 50 μL of the kit Luciferase Assay Reagent providing the D-Luciferin substrate into 96-well plate containing 10 μL of cell lysate per well. Photon emission was measured for a period of 10 seconds with a delay time of 2 seconds after injection of the substrate.

### Western blot analysis

Proteins from cultured cells were extracted by lysis in RIPA buffer (50 mM Tris, 150 mM NaCl, 5 mM EDTA, 0.5% sodium deoxycholic acid, 0.5% NP-40, 0.1% SDS) supplemented with a protease inhibitor cocktail (Complete, Roche Applied Science, Neuilly-sur-Seine, France). Once sonicated on ice, protein extracts were clarified by centrifugation for 15 minutes at 16 000*g *at 4°C and subjected to SDS-PAGE. Proteins were then transferred to polyvinylidene difluoride membranes (Immobilon, Millipore, Billerica, CA) by electroblotting at 4°C for 90 minutes at 90 V. The antibodies used for Western blot analysis were the OX34 mouse monoclonal antibody directed against rat CD2 (hybridoma kindly provided by Pr Martin Rowe, Birmingham) [8] and a purified polyclonal antibody raised in goats against recombinant firefly luciferase (Promega) [9]. Blots were incubated with a secondary peroxidase-conjugated antibody and chemiluminescent detection was done using the Immobilon Western Chemiluminescent HRP Substrate (Millipore). When required, primary antibodies were removed from the blotted membrane using the ReBlot Plus Strong Antibody Stripping Solution (Chemicon, Millipore) for 5 to 20 minutes at room temperature.

### Flow cytometry analysis and sorting of cells transduced by the recombinant constructs

Transfected cells were treated with 5% FBS in PBS for 30 minutes to block non-specific staining. They were then incubated with the OX34 monoclonal antibody (IgG2a), diluted 1:200 in PBS with 3% FBS, and with secondary polyclonal goat anti-mouse immunoglobulins labelled with phycoerythrin (Dako Denmark A/S, Glostrup, Denmark) diluted at 20 μg/ml in PBS with 3% FBS.

For cell sorting, about 5 millions cells were sorted using the MoFlo^® ^Flow Cytometer (Beckman-Coulter, Villepinte, France) with a gate of at least two logs of fluorescence intensity above the backround (non transfected control cells). Sorted cells (from 10 to 40%) were collected in 96 well-plates at a density in the range of 5000 to 50000 cells per well.

### Assessment of *in vitro *cell growth by MTT reduction assay

Daudi and HeLa cells were seeded in 96-well plates at a density of 25 000 and 5 000 cells, respectively, with 100 μl culture medium per well. An MTT (Thiazolyl Blue Tetrazolium Blue) reduction assay was used to assess the amount of viable cells. MTT powder (Sigma) was solubilized in PBS to make a stock solution at 250 mg/ml. 25 μl of this stock solution were added to each well subjected to this assay. After 4 h of incubation, 100 μl of solubilisation buffer (0.7 M SDS; 4.5 M dimethyl-formamide; 0.02N acetic acid; 1N HCl; pH 7.4) were added to each well to solubilize the formazan salt resulting from MTT reduction. The amount of product was evaluated by absorption at 550 nm on a spectrophotometer. Assessment of viable cells was performed just after seeding and for the next 3 days.

### Obtention of xenografted tumors

Eight-week-old female nude mice were anesthetized with 3% isoflurane. Prior to subcutaneous injection, 6.10^6 ^viable control or stably transfected Daudi cells were suspended in a mix of RPMI medium (100 μl) and BD Matrigel™ (100 μl) (growth factor reduced) (BD Biosciences, Le Pont de Calix, France).

### Bioluminescent imaging (BLI)

For *in vivo *imaging, mice were treated by intraperitoneal injection (150 mg/kg in PBS) of the D-luciferin substrate (Promega). The anesthetized mice were placed onto warmed stage inside the light-tight IVIS 50 box. In this study, mice were imaged fifteen minutes after D-luciferin injection to ensure consistent photon flux emitted during the oxidation of the substrate. The IVIS 50 camera system was used to visualize tumors, and photon measurement was defined around the tumor area and quantified using Living Image software (Xenogen for Caliper Life Science SA, Villepinte, France).

## Results

### Short-term expression of the tCD2-luciferase 2 chimera in 293 cells

**Figure 1:**
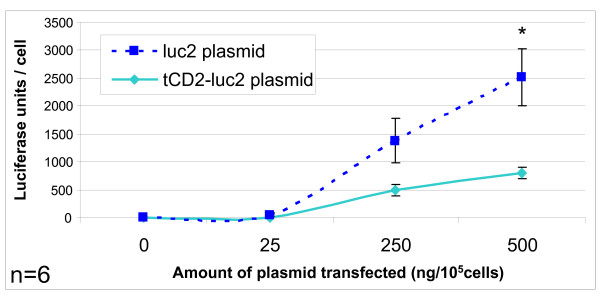
**Luciferase activity in 293 cells following short-term transfection of the wild-type *luciferase 2 *and *tCD2-luciferase 2 *genes**. Recipient epithelial cells (293) were seeded at 2.10^5^/well in 6-well plates and transfected using the Turbofect reagent (Fermentas) with increasing concentrations of plasmid DNA encoding either for the wild-type luc2 or the tCD2-luc2 chimera. They were grown *in situ *for 48 h prior to protein extraction and luciferase assay. For each condition, luciferase activity was normalized according to cell counts made on a fraction of the cells collected for the luciferase assay. This experiment was repeated six times (n = 6). Error bars represent SD of 6 experiments. Luciferase activities are significantly different for the two genes when using 500 ng of transfected plasmid (Mann&Whitney test, p < 0.05).

**Figure 2:**
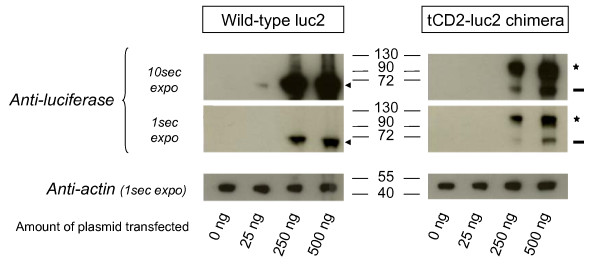
**Western blot analysis of luciferase 2 (luc2) and tCD2-luc 2 proteins in short-term transfected 293 cells**. The 293 cells were seeded and transfected as explained in the legend of Figure 1. They were grown *in situ *for 48 h prior to protein extraction and western blot analysis. The wild-type luciferase 2 and the tCD2-luc2 chimera are pointed out by an arrowhead and a star, respectively. An apparent cleaved fragment of the tCD2-luc2 protein with a size identical to the wild-type luciferase 2 is pointed out by a dash. The western blot membranes were restained with an anti-actin (Sigma) for loading control.

**Figure 3:**
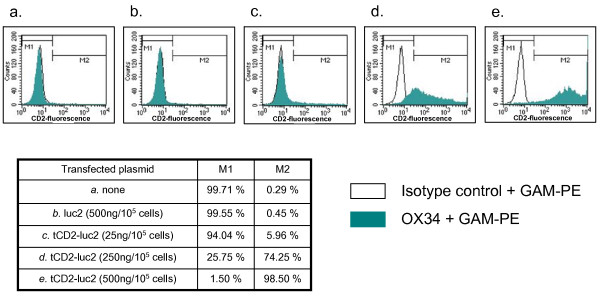
**Cell surface expression of the recombinant CD2 following short-term transfection of the *tCD2-luc 2 *gene in 293 cells**. The 293 cells were stained with the OX34 MAb reacting with a cell surface epitope of the rat CD2 protein. The M1 and M2 cell populations are defined according to the threshold of background fluorescence. For assessment of the background fluorescence, cells were first incubated with the mouse isotype control IgG2a (instead of the OX34) and then with the secondary antibody conjugated to phycoerythrin (goat anti-mouse-PE or GAM-PE).

A chimeric gene combining the first 232 codons of the rat *CD2 *(*tCD2*) to the full length gene encoding for luciferase 2 (*Photinus pyralis*) was inserted in a eukaryote expression plasmid under the control of the CMV promoter. In order to check that the chimeric gene was effectively translated in a protein, it was expressed in 293 cells by short-term transfection. Simultaneously, the wild-type luciferase 2 gene was transfected in the same cells using the same promoter in the same plasmid vector. A substantial luciferase activity was detected in cells transfected with the *tCD2-luciferase 2 *(*tCD2-luc2*) construct as well as the wild-type *luciferase 2 *(*luc2*) gene (Figure 1). In both cases, a substantial level of luciferase activity was obtained for concentrations of plasmids equal or lower than 250 ng/10^5 ^cells. Simultaneously the tCD2-luc2 and luc2 proteins were detected by western blot analysis in protein extracts of transfected cells using similar amounts of plasmids (about 250 ng/10^5 ^cells) (Figure 2). The tCD2-luc2 chimera was detected with even lower plasmid concentrations - in the range of 50 ng/10^5 ^cells - when staining its CD2 portion on the surface of live cells using the OX34 antibody and flow cytometry analysis (Figure 3).

### Long-term *in vitro *expression of the tCD2-luciferase 2 chimera in the context of lymphoid and epithelial cells

**Figure 4:**
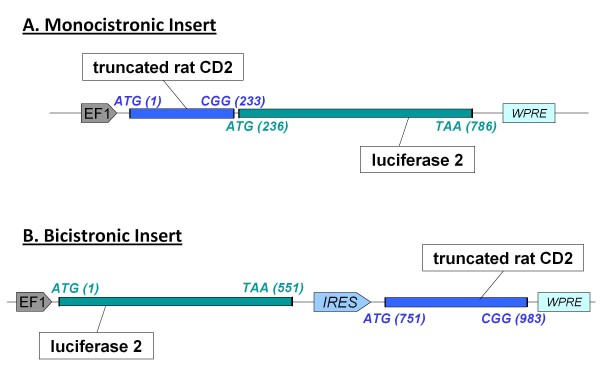
**Structure of monocistronic and bicistronic constructs inserted in lentiviral vectors for combined expression of the luciferase 2 and truncated rat CD2 proteins**. Lentiviral vectors were designed for expression of the luciferase 2 protein combined with a truncated form of the rat CD2 (tCD2) protein either as a fusion protein (monocistronic insert) or as two distinct proteins (bicistronic insert). Both types of constructs are inserted at an EcoR1 site in the pro-lentiviral plasmid under the control of the EF-1 alpha promoter and flanked by the WPRE sequence. Recombinant lentiviral particles were produced by tri-transfection in 293T cells as explained in the Materials and Methods section. A) The *tCD2-luc2 *insert contains the first 232 codons of the rat CD2 gene fused to 2 codons GAA (glutamic acid) and GTC (valine) added for cloning purpose (EcoR1 site) in frame with the full length luciferase 2 gene starting at codon 236. B) The bicistronic insert - *luc2-IRES-tCD2 *- contains the full length luciferase 2 gene linked by an IRES (Internal Ribosome Entry System sequence) to the truncated rat CD2 gene. This IRES (573 base pairs) was derived from the pQCXIX plasmid (Clontech).

**Figure 5:**
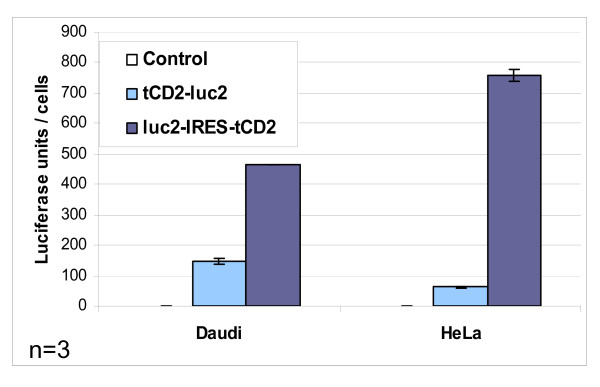
**Luciferase activity in stable lymphoid and epithelial transfectants carrying recombinant luciferase constructs**. Luciferase activity was assessed in protein extracts from Daudi and HeLa cells with stable expression of monocistronic (*tCD2-luc2*) or bicistronic (*luc2-IRES-tCD2*) luciferase constructs. The substrate was D-luciferin (Promega luciferase assay system, Promega). Light measurements were done on a Microlumat LB96P luminometer (Berthold, Thoiry, France). This experiment was repeated thrice; error bars represent SD of three experiments (n = 3). Error bars are not apparent above rectangles corresponding to Daudi *luc2-IRES-tCD2 *and Hela *tCD2-luc2 *because experimental data for these conditions were very consistent (very small error bars are not apparent for this size of the graph using the Microsoft Excel software).

**Figure 6:**
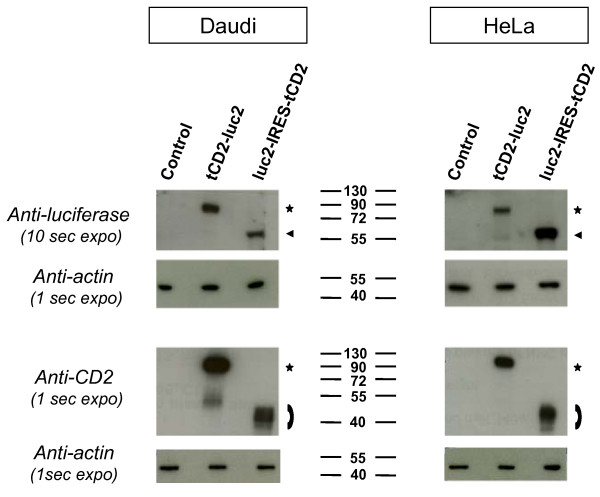
**Western blot analysis of recombinant luciferase and rat CD2 proteins in stable lymphoid and epithelial transfectants**. Protein extracts were prepared from stably transfected Daudi and Hela cells as well as from the corresponding control cells. After gel separation and membrane transfer, these extracts were analysed using antibodies directed to the luciferase protein or the rat CD2. The tCD2-luc2 chimera, the wild-type luciferase 2 and the truncated rat CD2 are pointed out by a star, an arrowhead and an arch, respectively. The western blot membranes were restained with an anti-actin for loading control. A minor cleaved fragment of the tCD2-luc2 protein with a size identical to the wild-type luciferase 2 is detected at a low level in the extract of Hela cells transfected with the *tCD2-luc2 *fusion gene.

**Figure 7:**
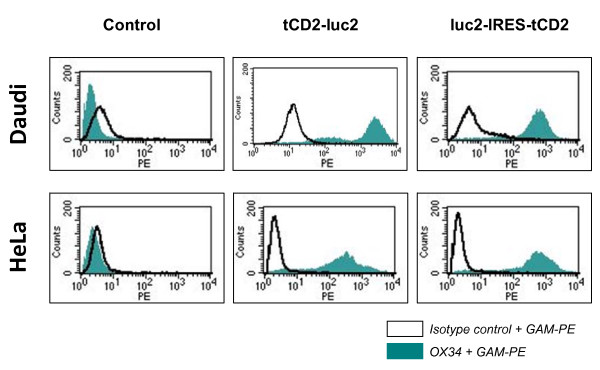
**Flow cytometry detection of cell surface recombinant CD2 in stable lymphoid and epithelial transfectants**. Stably transfected cells of lymphoid (Daudi) or epithelial (Hela) origin were stained with the OX34 MAb reacting with a cell surface epitope of the rat CD2 protein. The threshold of background fluorescence was defined according to cells incubated with the mouse isotype control IgG2a instead of the OX34, as explained for Figure 3.

Our next goal was to assess the expression level of tCD2-luc2 in stably transfected cells and its impact on cell viability. For this aim, the *tCD2-luc2 *fusion gene was inserted in a lentiviral vector under the control of the EF-1 alpha promoter (Figure 4, monocistronic construct). Simultaneously, another construct was built in order to compare the chimeric protein to the wild-type luciferase in a context of co-expression with the truncated rat CD2 but without fusion of these two species. For this aim, the wild-type luciferase gene driven by the EF1-alpha promoter was combined to the truncated CD2 gene driven by an Internal Ribosome Entry Site (IRES). This bicistronic construct called *luc2-IRES-tCD2 *was inserted in the same lentiviral vector as the one used for *tCD2-luc2 *(Figure 4, bicistronic construct). Both types of lentiviral particles were used to transduce lymphoid (Daudi) and epithelial (Hela) cells in order to make stable transfectants. In each of these four experimental conditions, more than 80% cells were transfected as shown by staining of the CD2 protein at the surface of live cells and flow cytometry analysis using the OX34 antibody (data not shown). CD2-positive cells were subsequently sorted by flow cytometry to get rid of the minority of untransfected cells. After expansion of sorted cells for at least two weeks, luciferase activity was repeatedly investigated in the extracts of Daudi and Hela cells (Figure 5). A significant activity was consistently detected in the *tCD2-luc2 *transfectants although not as intense as for *luc2-IRES-tCD2 *transfectants. Similar levels of luciferase activity were recorded in another epithelial cell line - MDA-MB-231 - stably transfected with the *tCD2-luc2 *construct (data not shown). As shown in Figure 6, the tCD2-luc2 and luc2 proteins detected by western blotting, were in the same range of concentrations in both types of stable transfectants, suggesting that the above-mentioned difference in luciferase activity was related to sub-optimal activity of the chimeric protein rather than to its weak production or rapid degradation. Like for short-term transfected 293 cells, a minor cleaved fragment of the tCD2-luc2 protein with a size identical to the wild-type luciferase 2 was detected in stably transfected Hela cells (Figures 2 and 6). However, the intact fusion protein was clearly predominant with anti-luciferase as well as anti-CD2 staining. Finally surface CD2 staining and flow cytometry analysis was performed after at least 4 weeks of continuous growth (Figure 7). Massive and homogeneous surface expression of CD2 was recorded for transfected Daudi and Hela cells. The mean fluorescence was slightly higher for the *tCD2-luc2 *compared to the *luc2-IRES-tCD2 *construct in Daudi cells whereas it was the opposite for Hela cells.

### Absence of adverse effects of the tCD2-luciferase 2 chimera on cell growth *in vitro*

**Figure 8:**
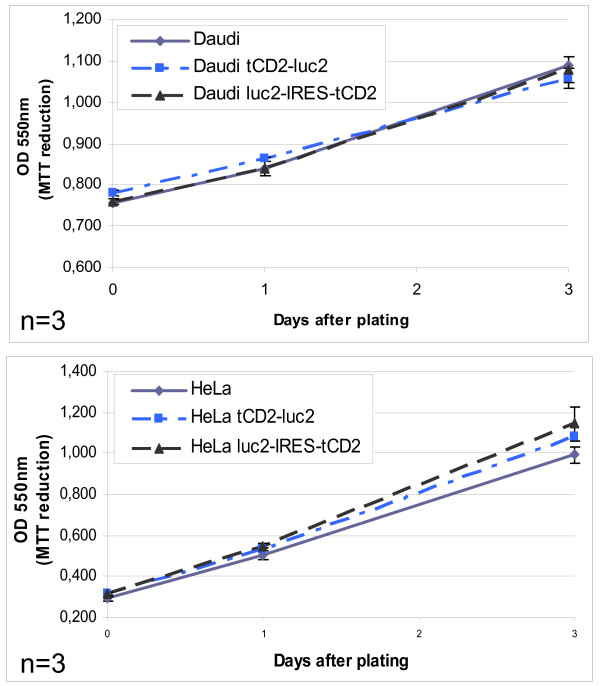
**Impact of the expression of luciferase constructs on lymphoid and epithelial cell growth *in vitro***. The viability and growth of stably transfected cells of lymphoid (Daudi) or epithelial (Hela) origin were tested in three day assays in 96-well plates (25000 and 5000 cells seeded per well at day 0 for Daudi and Hela cells, respectively). The amount of live cells per well was measured by an MTT reduction assay. Both experiments were repeated six times; error bars represent SD of 3 experiments (n=3).

Cell viability and growth assays were performed simultaneously on wild-type Daudi cells and their counterparts stably transfected with the *tCD2-luc2 *and *luc2-IRES-tCD2 *constructs. The amount of live cells was assessed in 96-well plates after one, two or three days of culture by an MTT-reduction assay. As shown in Figure 8, cell growth was identical in wild-type Daudi cells and both types of transfectants therefore providing no evidence of a toxic effect of the exogenous proteins, including the tCD2-luc2 chimera. The same observations were made for the transfected Hela cells using the same experimental methods (Figure 8).

### *In vivo *assessment of the tCD2-luciferase 2 chimera in xenografted tumors derived from Daudi cells

**Figure 9:**
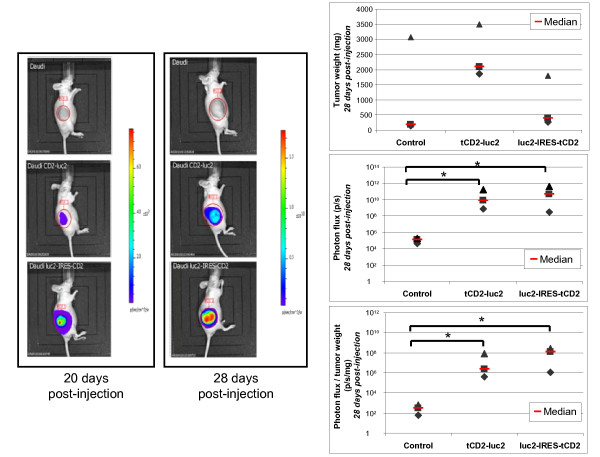
***In vivo *imaging of bioluminescent tumors containing two types of luciferase expression constructs**. Stably transfected Daudi cells (6.10^6 ^cells) were injected subcutaneously into the flank of nude mice forming visible tumors on average two weeks later. The resulting tumors were imaged after intra-peritoneal injection of the D-luciferin reagent using an IVIS 50 (Xenogen). Three series of three mice were imaged 20 and 28 days following injection of wild-type Daudi cells (used as controls, upper line of the left panel), Daudi cells transfected with the monocistronic luciferase construct (medium line) or the bicistronic construct (bottom line). At day 28, tumors were collected and weighted just after the imaging procedure. In the right panel, are plotted tumor weights (upper graph), total photon flux (medium graph) and the ratios of photon flux to tumor weights (bottom graph). The stars indicate a statistical difference from controls (Kruskal-Wallis one-way analysis, p < 0.06). Although in this experiment, injection of Daudi cells containing the *tCD2-luc2 *construct resulted in bigger tumors than the wild-type Daudi cells, this effect was not observed in another experiment (data not shown). These data as well as the results presented in this figure and in Figure 8 confirm that the tCD2-luc2 protein does not prevent cell growth *in vivo *as well as *in vitro*.

In order to prove that the cells expressing the tCD2-luc2 protein had appropriate luciferase activity for *in vivo *imaging, xenografted tumors were generated in nude mice by subcutaneous injections of wild-type, *tCD2-luc2 *and *luc2-IRES-tCD2 *transfected Daudi cells (three mice for each condition). After subcutaneous injection of 6.10^6 ^cells, tumors were visible as early as two weeks post-injection. Using an optical imaging system, substantial photon emission was recorded at days 20 and 28 post-injection for tumors carrying the *tCD2-luc2 *and the *luc2-IRES-tCD2 *constructs (Figure 9). Mice were sacrificed at day 28 just after assessment of bioluminescence, allowing immediate weight measurement for each tumor and its use as an index of tumor growth. There was a trend toward higher weights for tumors expressing the tCD2-luc2 chimera by comparison with the wild-type Daudi cells and cells expressing wild-type luciferase 2 from the *luc2-IRES-tCD2 *construct. This is a good indication that the tCD2-luc2 chimera is devoid of cell toxicity *in vivo *as well as *in vitro*. As shown on the bottom graph of Figure 9, although the average photon emission per mg of tumor tissue in tumors expressing the chimera is not as high as for the wild-type luciferase 2, it is well above the background emission recorded from control tumors (4 log difference).

## Discussion

The aim of this study was to obtain a system allowing rapid conversion of human tumorigenic cell lines into cell lines with stable luciferase expression, without antibiotic selection. This goal has been achieved by lentiviral transfection of two types of gene constructs: one is a monocistronic insert containing the chimeric *tCD2-luc2 *gene; the other one is a bicistronic insert resulting in concomitant expression of the wild-type luciferase 2 and the truncated rat CD2 as two distinct protein species. One advantage of the second strategy is to provide higher luciferase activity *in vitro *and *in vivo*. This is probably due to a sub-optimal enzymatic function of the chimeric luciferase. Insect luciferases are members of the acyl-adenylate/thioester-forming superfamily of enzymes [10]. They are evolutionary related to the fatty acyl-CoA synthetases which in physiological conditions are located in the outer mitochondrial membranes. The initial reaction catalyzed by firefly luciferases on their luciferin substrate is the formation of luciferase-bound luciferyl-adenylate in the presence of Mg++ and ATP. There are two options for the second step. Either oxygenation of luciferin resulting at the final stage in photon emission, release of AMP and CO_2_. Or formation under anaerobic conditions of a thio-ester bound between luciferin and co-enzyme A (L-CoA). These second step reactions are mutually exclusive. One can speculate that fusion of luciferase 2 to tCD2 changes its sub-cellular distribution and/or impairs its folding pattern [11]. As a consequence, formation of L-CoA might be enhanced at the expenses of luciferin oxygenation or both reactions might be inhibited. This issue will require further investigations to determine the subcelullar distribution of tCD2-luc2 chimera in the internal membrane network and if possible its secondary and tertiary structures. It remains that the bicistronic insert *luc2-IRES-tCD2 *might be a better choice when a very high sensitivity is required, for example for detection of very small lung metastases. However, on the long term, we favor the fusion protein strategy because two functions are achieved in a unique cistron: presentation of a membrane epitope for cell sorting and bioluminescence. In a multi-cassette expression vector, this compaction of two functions in a single protein will leave room for other inserts coding for proteins or transcripts of interest.

Previous studies have shown that N-terminal or C-terminal luciferase fusion proteins can be produced *in vivo *retaining luciferase enzymatic activity [12,13]. However both types of fusion proteins were GFP-luciferase proteins. To our knowledge, we are the first to report a chimeric protein combining the luciferase moiety with a type I membrane protein. We demonstrated that the tCD2-luc2 protein retains luciferase activity whereas its CD2 portion is presented at the surface of the plasma membrane. This applies to various cell types both in short and long term transfectants. In addition, our data suggest that the tCD2-luc2 protein do not induce significant cytotoxic effects *in vitro *as well as *in vivo*.

One important aspect of our experimental system is the use of recombinant VSV-G pseudotyped lentiviruses which can infect almost any cell type, both dividing and non-dividing. Lentiviral infection results in reverse transcription of the viral RNA genome and synthesis of a double strand proviral DNA which is inserted in the cellular genome by the viral integrase. Last generation self-inactivating lentiviruses exclude genes for viral genome replication and virion production, not only providing safety for use, but also stabilizing the integrated gene. These features enable simultaneous stable gene delivery in a large fraction of recipient cells thus allowing flow cytometry or bead capture selection instead of drug selection [14]. Another important characteristic of our system is the choice of a cellular house-keeping gene promoter with mild activity, the promoter of the EF-1α gene encoding for the human elongation factor-1α. According to previous studies such promoters are better suited to ensure sustainable and consistent activity of the transfected genes [12,15].

One alternative to bioluminescent imaging (BLI) is optical imaging based on a fluorescent protein expressed by tumor cells, for example GFP (λ _emission _= 510 nm) or red fluorescent proteins such as tdTomato (λ _emission _= 581 nm) or mCherry (λ _emission _= 610 nm) [16]. One major advantage of fluorescence-based imaging is that it does not require substrate injection but it has a major limitation due to high auto-fluorescence of animal tissues resulting in a poor signal to noise ratio. In contrast when adequate substrate concentration is attained bioluminescence provides sensitive detection with unsurpassed signal to noise ratios due to a lack of autobioluminescence [4]. However direct flow cytometry sorting of luciferase-transfected cells is not possible in routine conditions. One way to overcome this problem is to produce tumor cells combining expression of luciferase with expression of a fluorescent protein like GFP or tdTomato using fusion genes or bicistronic constructs [12,13,17] However our system of combined expression of luciferase and truncated CD2 could appear as an interesting alternative tool in two circumstances. First, some cell types do not tolerate GFP expression even at low concentrations. Its toxic effects have been described in non-malignant and malignant cells as well [18,19]. These toxic effects are, at least in part, related to inhibition of poly-ubiquitination [19]. They are probably underreported; in our own experience we have observed that some human nasopharyngeal carcinoma cell lines do not tolerate even low concentrations of GFP (Vicat et al., 2003 and unpublished observations) [20]. Since many fluorescent proteins were derived from GFP, they might not be tolerated in some cellular backgrounds as well. In such contexts, our tCD2-luciferase tool might provide a useful alternative. The same applies to *in vivo *experiments involving tumor targeting agents which are fluorescently labeled in order to assess their effective tumor homing, for example fluorescent nanoparticles, antibodies or antisens RNAs [21]. In these situations, it might be better to deal with non-fluorescent luciferase-positive tumor cells instead of using multiple chromophores.

## Conclusions

We here report the construction and characterization of a chimeric protein linking the N-terminus of the full-length luciferase protein to a truncated form of the rat CD2 protein. We demonstrate that the chimeric protein achieves plasma membrane insertion while retaining luciferase activity. Long-term, stable expression of this chimera is readily obtained in various human tumor cell lines by lentiviral transduction combined to flow cytometry sorting using an antibody directed to the rat CD2. Drug selection of tumor cells is not required for use of this experimental system. It is expected to be useful for experiments where stable GFP, labelling of tumor cells has to be avoided. The tCD2-luc2 protein also opens interesting perspectives for use in multi-cassette vectors, for example vectors containing one cassette coding for a shRNA combined to a cassette coding for a tracer protein.

## List of Abbreviations Used

EGF: Epidermal Growth Factor; CMV: Cytomegalovirus; RIPA: Radio Immuno Precipitation Assay; HRP: HorseRadish Peroxidase; EF-1 alpha: elongation factor-1 alpha; WPRE: woodchuck hepatitis virus posttranscriptional regulatory element; LTR: Long Terminal Repeat sequence; BLI: Bioluminescent Imaging; GFP: Green Fluorescent Protein

## Authors' contributions

ASJ and MG have made plasmid constructions, cell transfections, lentiviral transductions, protein expression studies *in vitro *and *in vivo*. CB and CG have been involved in animal works. MW has shared his expertise about xenografted tumor models. PB has designed the study with ASJ and CG. ASJ drafted the manuscript which was corrected and completed by PB. All authors read and approved the final manuscript.
